# College Notes

**Published:** 1893-05

**Authors:** 


### College Notes.

University of Buffalo, Medical Department—Alumni Association.—The annual meeting of the Alumni Association of the Medical Department of the University of Buffalo, will be held on Commencement Day, Tuesday, May 2, 1893. The meeting will be called to order at 10.30 A. M., in Alumni hall of the University building, 24 High street, to listen to the address of welcome, and to transact such business as may properly come before it. At 2.00 o'clock P. M. the association will again assemble to listen to the address of the President and the scientific programme. At 7.30 o'clock P. M. the members and their friends are invited to be present at the commencement exercises to be held in Music Hall.

The annual banquet will be held at the Tifft, at 10.00 o'clock P. M. Members can obtain tickets of the Executive Committee, and it is earnestly urged upon each alumnus to attend, and to contribute to the success of our annual meeting.

The Executive Committee entreats all alumni to enroll themselves as members of this association. The remarkable advancement and success of the university is a benefit to each alumnus, and he should still further show his loyalty to his alma mater by sustaining the good work, by his attendance, his deeds, and his small contributions.

The annual dues ($1.00) should be promptly transmitted or presented to the treasurer, in order that the necessary expenses of the meeting may be honorably met.

### ORDER OF EXERCISES.

*Morning Session.*—Address of Welcome, Prof. Roswell Park. Business session. Election of officers.

*Afternoon Session.*—The President's Annual Address. Papers and Discussion. The Influence of Marriage on Disease of the Female Sexual Organs, Dr. Paul F. Mundé ; The Causative Relation of the Minor to the Major Gynecological Diseases, Dr. C. C. Frederick ; The Careless Examination of Urine, Dr. Frederick Stanbro ; Remarks on Operative Surgery of the Rectum, Dr. Edward Clark.

At the commencement exercises in the evening, the address to the graduating class will be given by Dr. Paul F. Mundé, of New York.

### ALUMNI ASSOCIATION—OFFICERS, 1892–'93.

President, E. R. Hopkins, '77, Silver Creek, N. Y.; First Vice-President, W. C. Phelps, '66, Buffalo, N. Y.; Second Vice-President, Horace Hoyt, '48, E. Aurora, N. Y.; Third Vice-President, Ernest Wende, '78, Buffalo, N. Y.; Fourth Vice-President, E. H. Woolsey, '68, Oakland, Cal.; Fifth Vice-President, P. W. Van Peyma, '72, Buffalo, N. Y.; Permanent Secretary, John J. Walsh, '71, Buffalo, N. Y.; Recording Secretary, F. B. Willard, '90, Buffalo, N. Y.; Treasurer, H. U. Williams, '89, Buffalo, N. Y.

*Trustees.*—Henry Lapp, '65, Clarence, N. Y.; F. E. L. Brecht, '72, Buffalo, N. Y.; E. C. W. O'Brien, '67, Buffalo, N. Y.; Joseph Fowler, '73, Buffalo, N. Y.; Mahlon B. Folwell, Buffalo, N. Y.

*Executive Committee.*—William H. Bergtold, '86 ; Allen A. Jones, '89 ; Albert H. Briggs, '71 ; E. R. Hopkins, President Alumni Association (ex-officio) ; John Parmenter, Secretary Medical Faculty.

---

THE NIAGARA UNIVERSITY, MEDICAL DEPARTMENT—ANNUAL MEETING.—Commencement exercises of the Medical Department of Niagara University will be held on Thursday, May 4, 1893. The programme outlined by the committee is as follows : The Board of Medical Examiners of Niagara University will examine the candidates for graduation at ten o'clock in the morning. At 10.30 A. M., the exercises of the Alumni Association will begin in the lecture room of the college on Ellicott street. Address of Welcome, Professor Alvin A. Hubbell ; President's Address, Clarence B. LeVan, M. D.; Business Session ; Election of Officers.

*Afternoon Session.*—Papers and Discussion ; Causes and Treatment of Chronic Gonorrhea, J. H. Dowd, M. D., Buffalo, N. Y.; Diarrheas of Children, Edward Torry, M. D., Allegany, N. Y.; Treatment of Chronic Ulcers of the Leg, L. J. McAdam, M. D., Buffalo, N. Y.

At the commencement exercises in the evening, to be held at the Star Theatre, [illegible] 7.30 o'clock, Dr. Thomas Lothrop will deliver the decennial ad[illegible]ess, and the Rev. Thos. Slicer will address the graduates. Th[illegible] annual banquet will be held at 10.00 o'clock, P. M.

### ALUMNI ASSOCIATION—OFFICERS FOR 1892–'93.

Clarence B. LeVan, M. D., President, Buffalo, N. Y.; Edgar A. Forsyth, M. D., First Vice-President, Buffalo, N. Y.; Alfred M.

Bayliss, M. D., Second Vice-President, Buffalo, N. Y.; William H. Norrish, M. D., Secretary, Buffalo, N. Y.; John J. Twohey, M. D., Permanent Secretary, Buffalo, N. Y.; E. Eugene Martin, M. D., Treasurer, Buffalo, N. Y.; John M. Hewitt, M. D., Buffalo, N. Y., Albert J. Colton, M. D., Buffalo, N. Y., Linus J. McAdam, M. D., Buffalo, N. Y.; Clarence B. LeVan, President Alumni Association (ex-officio) ; Alvin A. Hubbell, M. D., Secretary Medical Faculty (ex-officio), Executive Committee.

